# Apolipoprotein epsilon 3 alleles are associated with indicators of neuronal resilience

**DOI:** 10.1186/1741-7015-10-35

**Published:** 2012-04-13

**Authors:** Orwa Aboud, Robert E Mrak, Frederick Boop, Sue T Griffin

**Affiliations:** 1 Donald W. Reynolds Department of Geriatrics, Reynolds Institute on Aging, 629 Jack Stephens Drive, Little Rock, AR 72205, USA; 2 Geriatric Research Education Clinical Center, Central Arkansas Veterans Healthcare Systems, 4300 West Seventh Street, Little Rock, AR 72205, USA; 3 Department of Neurobiology and Developmental Sciences, University of Arkansas College of Medicine, 4300 Markham Street, Little Rock, AR 72205, USA; 4 Department of Pathology, University of Toledo Health Sciences Campus, 3000 Arlington Avenue, Toledo, OH 43614, USA; 5 Department of Neurosurgery, University of Tennessee Health Sciences Center, 50 N Dunlap St, Memphis, TN 38163 USA

**Keywords:** Amyloid beta (Aβ), Alzheimer disease, *APOE *genotype, DNA damage, epilepsy, interleukin-1, neuroinflammation, phosphorylated tau, synaptophysin, TUNEL

## Abstract

**Background:**

Epilepsy is associated with precocious development of Alzheimer-type neuropathological changes, including appearance of senile plaques, neuronal loss and glial activation. As inheritance of *APOE ε4 *allele(s) is reported to favor this outcome, we sought to investigate neuronal and glial responses that differ according to *APOE *genotype. With an eye toward defining ways in which *APOE ε3 *alleles may foster neuronal well-being in epilepsy and/or *APOE ε4 *alleles exacerbate neuronal decline, neuronal and glial characteristics were studied in temporal lobectomy specimens from epilepsy patients of either *APOE ε4,4 *or *APOE ε3,3 *genotype.

**Methods:**

Tissue and/or cellular expressions of interleukin-1 alpha (IL-1α), apolipoprotein E (ApoE), amyloid β (Aβ) precursor protein (βAPP), synaptophysin, phosphorylated tau, and Aβ were determined in frozen and paraffin-embedded tissues from 52 *APOE ε3,3 *and 7 *APOE ε4,4 *(0.25 to 71 years) epilepsy patients, and 5 neurologically normal patients using Western blot, RT-PCR, and fluorescence immunohistochemistry.

**Results:**

Tissue levels of IL-1α were elevated in patients of both *APOE ε3,3 *and *APOE ε4,4 *genotypes, and this elevation was apparent as an increase in the number of activated microglia per neuron (*APOE *ε*3,3 *vs *APOE ε4,4 *= 3.7 ± 1.2 vs 1.5 ± 0.4; *P *< 0.05). This, together with increases in βAPP and ApoE, was associated with apparent neuronal sparing in that *APOE ε4,4 *genotype was associated with smaller neuron size (*APOE ε4,4 *vs *APOE ε3,3 *= 173 ± 27 vs 356 ± 45; *P *≤ 0.01) and greater DNA damage (*APOE ε4,4 *vs *APOE ε3,3 *= 67 ± 10 vs 39 ± 2; *P *= 0.01). 3) Aβ plaques were noted at early ages in our epilepsy patients, regardless of *APOE *genotype (*APOE ε4,4 *age 10; *APOE ε3,3 *age 17).

**Conclusions:**

Our findings of neuronal and glial events, which correlate with lesser neuronal DNA damage and larger, more robust neurons in epilepsy patients of *APOE ε3,3 *genotype compared to *APOE ε4,4 *genotype carriers, are consistent with the idea that the *APOE *ε*3,3 *genotype better protects neurons subjected to the hyperexcitability of epilepsy and thus confers less risk of AD (Alzheimer's disease).

Please see related article: http://www.biomedcentral.com/1741-7015/10/36

## Background

Epilepsy is associated with precocious development of Alzheimer-type neuropathological changes, and the *APOE ε4 *genotype has been associated with further risk of development of such changes [1,2]. A role for glial activation with excess expression of cytokines in epilepsy pathogenesis was first recognized as enlargement of microglia and astrocytes with overexpression of IL-1 and S100B, respectively [3-5]. Such findings gave rise to a new understanding of the role of glial activation and overexpression of cytokines as potential precursors of neurodegenerative change, including Aβ plaques and neurofibrillary tangles [6]. These findings are consistent with the idea that glia-related neuroinflammatory events are early contributors to epilepsy pathogenesis.

Neuronal stress, such as the hyperexcitability induced by glutamate in epilepsy, elevates neuronal expression of βAPP and release of sAPP, which activates microglia and induces excess IL-1 production. This elevation in IL-1 production is attenuated by ApoE 3, but not ApoE 4 [7]. In turn, IL-1 induces further neuronal expression of βAPP and sAPP leading to further microglial activation and further release of IL-1 [8]. IL-1 also induces neuronal expression of ApoE [9], which in turn induces further expression of βAPP in an ApoE isoform-dependent manner; with ApoE3 more effective than ApoE4 [10].

A great deal of research has been dedicated to understanding how and why the presence of an *APOE ε4 *allele(s) is so strongly associated with negative outcomes in neurological conditions, such as head injury [11]. Here, rather than taking this tack, we chose to investigate the potential for beneficial effects conferred by *APOE ε3 *alleles due to their neuroprotective potential. Tissue samples from temporal lobes resected from epilepsy patients carrying two *APOE ε3 *alleles were examined regarding an association between inheritance of these alleles and determinants of neuronal resilience. These determinants included the ability of neurons to mount appropriate acute phase responses, including increases in βAPP and ApoE, as well as management of DNA damage, maintenance of morphological integrity and glial activation. Our findings indicate that the *APOE ε3,3 *genotype confers a neuroprotective advantage over the *APOE ε4,4 *genotype, in the setting of intractable epilepsy with its accompanying hyperexcitability-induced neuronal damage, glial activation and excessive expression of the proinflammatory cytokine IL-1α.

## Methods

### Patients and specimens

Resected temporal lobe tissues were obtained from 95 epilepsy patients; of those 59 were included in this study (39 males and 20 females; 52 *APOE ε3,3 *and 7 *APOE ε4,4*) with an age at surgery ranging from 0.25 to 71 years. Analyses of surgical waste remains from temporal lobectomy surgeries to treat intractable, drug-resistant epilepsy were compared to those of autopsy samples from neurologically normal individuals brought to autopsy for reasons other than this study. Both surgical waste and autopsy tissue are exempt under 46.101 5(b) and approved by our University of Arkansas Institutional Review Board.

All patients underwent anterior temporal lobectomy for treatment of medication-resistant intractable epilepsy. Tissue was sectioned at 4 mm intervals and alternate sections were preserved by flash freezing for molecular analyses and by formalin fixation for histological evaluation. Preliminary immunohistochemical analysis was performed on all epilepsy cases, and a smaller group was selected for further investigation. Six *APOE ε3,3 *cases (five males and one female, ages 18, 24, 38, 44, 67 and 57 years, respectively) and four *APOE ε4,4 *cases (three males and one female, ages 10, 22, 50 and 34, respectively) were selected for more extensive analyses, based on age in the case of *APOE ε3,3 *patients, and with regard to availability of sufficient frozen tissue for molecular analyses among *APOE ε4,4 *patients. Sufficient frozen tissue and fixed tissue was available for both immunohistochemical and molecular analyses of four *APOE ε4,4 *patients (three males and one female, ages as above). For uniformity, immunohistochemical examination was restricted to cortical layers III, IV, V and VI of the superior temporal lobe. For comparison of results from our *APOE ε3,3 *and *APOE ε4,4 *genotype patients, analogous temporal lobe tissues from neurologically normal individuals of varying *APOE *genotype and at older ages (four males and one female, ages 71, 97, 59, 50 and 93 years) were assessed. This selection was based on the premise that individuals with pre-AD (Alzheimer's disease) or with AD at these ages would have plaques.

### Reagents

The antibodies used were as follows: rabbit anti-human IL-1α (Peprotech, Rocky Hill, NJ, USA, 4:1,000); goat anti-human APOE (Life Technology, Grand Island, NY, USA, 1:50); mouse anti-human Aβ/βAPP (Covance, Denver, CO, USA, 1:1,000); rabbit anti-synaptophysin (Abcam, Cambridge, MA, USA, 1:1,000); rabbit anti-phosphorylated tau (Abcam 1:3,000); rabbit anti-actin (Santa Cruz Biotechnology, Santa Cruz, CA, USA, 1:5,000) were diluted in antibody diluent (DAKO, Carpenteria, CA, USA), and Iba-1 (WAKO, Richmond, VA, USA,1:400). Mounting media containing Prolong Gold antifade reagent with DAPI (Life Technologies) was used to stain nuclei.

### Immunohistochemistry

Paraffin-embedded tissue was sectioned at 7 μm, deparaffinized in xylene and rehydrated in graduated ethanol solutions to deionized water. Sections for IL-1α immunoreaction were placed in boiling sodium citrate buffer (0.01 M, pH 6.0) for 20 minutes; sections for βAPP and ApoE were placed in trypsin solution for 10 minutes at 37°C, and all were blocked using protein block (DAKO), and incubated overnight at room temperature. Secondary antibodies, Alexa Fluor donkey anti-goat and donkey anti-rabbit were diluted in antibody diluent (DAKO) and sections were incubated for 60 minutes, washed three times for 5 minutes each in distilled H_2_O, and coverslipped with prolong Gold with DAPI.

### Plaque analysis

Plaques were identified by the simultaneous presence of ApoE and Aβ immunoreactivity. The number of plaques in 10 consecutive 20X images (0.37 mm^2^) from sections of tissue from each patient was enumerated. Plaque phase was based on Braak and Braak staging of Aβ plaques [12] and estimated with regard to our experience with such estimation in Alzheimer tissue.

### Image analysis

Similar to a previous study [10], a quantitative approach was used to examine the number of glia and neurons. Three images per slide (40X magnification) were captured at identical exposure settings, using a Nikon Eclipse E600 microscope (Melville, NY, USA) equipped with a Coolsnap ES monochrome camera (Photometrics, Tucson, AZ, USA). Each of the three images, spanning 37,241.5 μm^2^, was acquired and analyzed using NIS-Elements BR3 software http://Nikon.com and thresholded. Only microglia immediately adjacent to neuron somas were counted. Data were analyzed by ANOVA to assess difference among groups. Significance was provided by *P *≤ 0.05.

### Reverse transcription (RT) reaction and polymerase chain reaction (PCR) amplification

Total RNA was extracted from brain tissue using TriReagent™ RNA (Molecular Research Center, Cincinnati, OH, USA) according to the manufacturer's instructions. RT-PCR was performed as previously described [9]. Briefly, for comparisons of mRNA levels among different RNA samples, RT reactions were performed simultaneously using reagents from a single master mix. PCR was performed using reagents from Clontech (Mountain View, CA, USA). The sequences of primers for human IL-1α and GAPDH, amplification cycles and annealing temperature are provided in Table 1. PCR reactions were stopped by incubation for 10 minutes at 72°C. Equal volumes of reaction mixture from each sample were loaded onto 1.2% agarose gels, and fluorescent images were digitally captured for analysis of intensity with NIH Image software 1.60 version http://rsbweb.nih.gov/nih-image/. Levels of IL-1α were normalized relative to GAPDH in the same sample.

**Table 1:** Human gene sequences for IL-1α and GAPDH, PCR annealing temperatures and number of amplification cycles

Gene analyzed	Human sequences	Annealing temp. (°C)	**Cycle no**.
Gel-based PCR			

IL-1α	F: AAG CCT TCC TGC CGC AAC	57	32
			
	R: CTG CAC CTA CCA AAC ACG G		

GAPDH	F: AGG TCG GAG TCA ACG GAT TTG	57	32
			
	R: TGG CAG GTT TTT CTA GAC GGC		

### Western immunoblot assay

Proteins were extracted from brain tissue in a lysis buffer comprising 20 mM Tris-HCl (pH 7.5), 150 mM NaCl, 1% Nonidet P40, 1 mM EGTA, 1 mM EDTA and 1% sodium deoxycholate; lysates were quantified using a Micro BCA assay reagent kit (Pierce, Rockford, IL, USA) as described previously [13]. Aliquots (50 μg each) were loaded onto 4 to 12% Criterion^XT ^precast Gels (Biorad, Hercules, CA, USA, Catalog # 345-0123), subjected to electrophoresis at 90 V for 1.5 h, and transferred to nitrocellulose membranes. Blots were blocked in I-Block Buffer (Applied Biosystem Inc., Bedford, MA, USA) for 60 minutes, then incubated overnight at 4°C with either goat polyclonal antibody anti-IL-α (Santa Cruz Biotechnology 1:500), mouse anti-human Aβ/βAPP (Covance 1:1,000), rabbit anti-synaptophysin (Abcam 1:1,000), rabbit anti-phosphorylated tau (Abcam 1:3,000), or rabbit anti-actin (Santa Cruz Biotechnology 1:5,000); the latter of which was used here for calculating the relative levels of the other proteins assessed by western blot analyses. Membranes were then incubated for 1 h at room temperature with alkaline phosphatase-conjugated secondary antibody and developed using the Western-Light™ Chemiluminescent Detection System (Applied Biosystem Inc., Bedford, MA, USA). Autoradiographs were digitized and analyzed using NIH Image software, version 1.60.

### TUNEL staining procedure

For terminal deoxynucleotidyl transferase dUTP nick end labeling (TUNEL) (NeuroTacs Kit, 4823-30-K, Trevigen, Gaithersburg, MD, USA) reactions, rehydrated sections were permeablized with NeuroPore^® ^for 30 minutes at room temperature, washed in PBS buffer, placed in TdT labeling buffer for 5 minutes, treated with the labeling reaction mix (TdT dNTP, 50 × Mn^+2^, and TdT Enzyme) for 60 minutes at 37°C followed by stop buffer for 5 minutes, then streptavidin AF 594 conjugate (Invitrogen, S32356) for 10 minutes at room temperature. The sections were then treated with 0.1% Sudan black B in 70% ethanol for two minutes to block lipofuscin autofluorescence, washed in three changes of distilled H_2_O, five minutes each; and coverslipped with Prolong Gold with DAPI.

### Statistical analysis

Data were analyzed using an unpaired *t*-test, and values were considered significantly different when the *P*-value was ≤ 0.05. Results are expressed as mean ± SD.

## Results

### Numbers of IL-1α-immunoreactive microglia per neuron are *APOE *genotype-dependent

**Figure 1:**
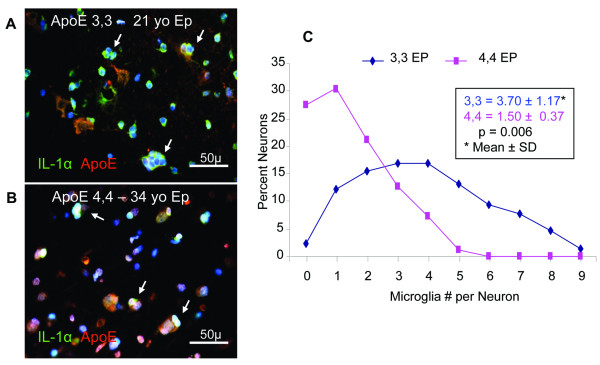
**Microglial numbers per neuron in epilepsy relative to APOE genotype IL-1α-immunoreactive microglia (green) clustered around individual ApoE-immunoreactive neuron soma (red) in a 21-year-old *APOE ε3,3 ***(A) **and a 34-year-old *APOE ε4,4 ***(B) **patient**. A maximum of 8 microglia per neuron were present in *APOE ε3,3 *patients compared to four or fewer in *APOE ε4,4 *patients **(C)**. Overall, *APOE ε3,3 *patients had twice as many microglia per neuron as did *APOE ε4,4 *patients. Nuclei were stained with DAPI (blue). White arrows denote neurons with microglia.

An initial screening of 59 epilepsy patients (52 *APOE ε3,3*; 7 *APOE ε4,4*) and 5 neurologically normal controls revealed an observable elevation in numbers of microglia per neuron soma in brain tissue from epilepsy patients compared to that in brain tissue from controls (data not shown). A smaller group of age-matched patients with either *APOE ε3,3 *(n = 6) or *APOE ε4,4 *(n = 4) genotype was chosen for further analysis to determine if this increase in numbers of IL-1α immunoreactive microglia (Iba-1-immunoreactive) per ApoE-immunoreactive neuron segregated according to *APOE *genotype. Numbers as high as 8 IL-1α/Iba-1-immunoreactive microglia were noted clustered immediately adjacent to individual ApoE-immunoreactive neurons in *APOE ε3,3 *patients (Figure 1A) compared to a maximum of three microglia per neuron in *APOE ε4,4 *patients (Figure 1B). Overall, *APOE ε3,3 *neurons had twice as many associated microglia as did *APOE ε4,4 *neurons (3.7 ± 1.2 vs 1.5 ± 0.4; *P *< 0.01) (Figure 1C). As to the total number of microglia per 0.037 mm^2 ^in tissue from epilepsy patients with *APOE ε3,3 *vs *APOE ε4,4 *genotype (37.2 ± 10.6 vs 23 ± 5.7; *P *< 0.05).

### Neuron number and size with regard to *APOE *genotype

**Figure 2:**
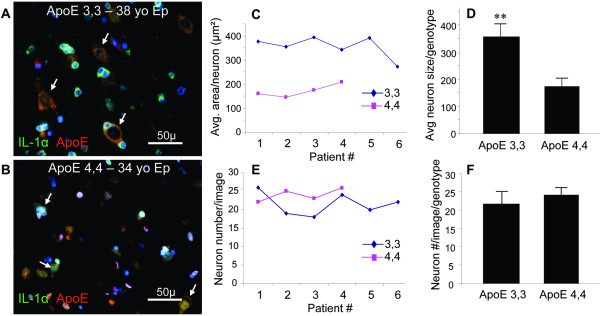
**Neuron size and number in epilepsy relative to APOE genotype An apparent larger size of neurons (arrows) in patients with *APOE ε3,3 ***(A) **relative to those in patients with *APOE ε4,4 ***(B) **genotype was confirmed by computer assisted area measurements of individual neurons (*APOE ε3,3 *= 356 ± 45 μm^2 ^vs *APOE ε4,4 *= 173 ± 27 μm^2^; *P *< 0.001) (C and D)**. However, the numbers of neurons per image were similar for both genotypes (*APOE ε3,3 = *21.5 ± 3.1 vs *APOE ε4,4 = *24.0 ± 1.8; *P *= 0.18) **(E and F)**.

To assess the consequences of epilepsy and *APOE *genotype on neurons, we counted neurons in cortical layers III to VI of three specific areas of the temporal lobe. By histological inspection, changes in the appearance of individual neurons were evident, with the distinguishing feature being an *APOE ε4,4 *genotype-associated decrease in size of both the cytoplasm and nucleus (Figure 2A, B). When quantified, this general impression correlated with a genotype-related difference in neuronal size. The average size of neurons in patients with *APOE ε4,4*, was smaller than in patients with *APOE ε3,3 *(173 ± 27 vs 356 ± 45 μm^2^; *P *< 0.001) (Figure 2C, D), but the numbers of neurons were not different in cortical layers of patients with *APOE ε4,4 *and *APOE ε3,3 *genotypes (24 ± 2 vs 22 ± 3; *P *= 0.18) (Figure 2E, F),.

### Neuronal DNA damage is related to *APOE *genotype

**Figure 3:**
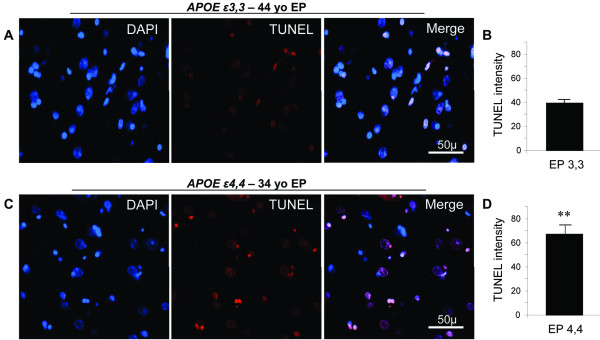
**Neuronal DNA damage in epilepsy relative to APOE genotype Neurons with nuclear DNA damage were present in similar numbers in analogous areas of temporal lobe for both *APOE *genotypes (A and C)**. However, the extent of damage per neuron, as assessed by semi-quantitative analysis of TUNEL fluorescence intensity (arbitrary units, au) in these areas, was greater in patients with *APOE ε4,4 *than in those with *APOE ε3,3 *genotype (*APOE ε4,4 *= 67.1 ± 9.5 vs *APOE ε3,3 *= 39.2 ± 2.3; *P *= 0.01) **(B and D)**. TUNEL labeling (red) and DAPI nuclear staining (blue).

DNA damage was present in similar numbers of neurons in analogous areas of the temporal lobe, without regard to *APOE *genotype (Figure 3A, C). However, the extent of damage per neuron, as assessed by TUNEL fluorescence intensity, was greater in patients with *APOE ε4,4 *than with *APOE ε3,3 *genotype (67 ± 10 vs 39 ± 2; *P *= 0.01) (Figure 3B, D).

### βAPP tissue levels as a function of IL-1α expression and *APOE *genotype

**Figure 4:**
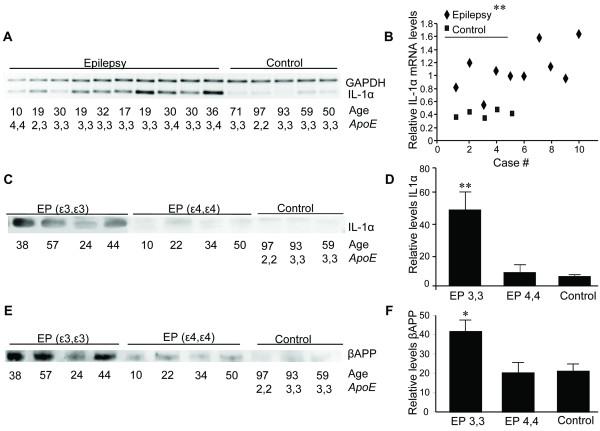
**Neural cell acute phase responses in epilepsy relative to APOE genotype IL-1α mRNA levels in temporal lobe tissue from epilepsy patients were elevated compared to levels in control patients (A)**. With the exception of one patient with *APOE ε4,4 *genotype, patients with epilepsy had mRNA levels that were greater than those of control patients **(B)**. By Western blot **(C)**, the relative levels of IL-1α protein from epilepsy patients with *APOE ε3,3 *were four-fold higher (51 ± 29) than those from either epilepsy patients with *APOE ε4,4 *genotype (8.6 ± 4.3) or those of neurologically normal controls (6.3 ± 1.2; *P *= 0.01 for all comparisons) **(D)**. These elevations in both IL-1α mRNA and protein levels were accompanied by an increase in βAPP expression **(E) **that was limited to *APOE ε3,3 *patients (*APOE ε3,3 *= 42 ± 11 vs *APOE ε4,4 *= 20 ± 11 and control = 21 ± 7; *P *= 0.03 for all comparisons) **(F)**.

The increased numbers of microglia per neuron noted in *APOE ε3,3 *vs *APOE ε4,4 *patients were reflected at the level of IL-1α synthesis and production. Overall, levels of IL-1α mRNA were elevated within a group of 10 of our epilepsy patients with various combinations of *APOE ε2, APOE ε3*, or *APOE ε4 *alleles compared to levels in control patients (Figure 4A), although one patient with *APOE ε4,4 *genotype had mRNA levels that were not different from that of control patients (Figure 4B). Overall, patients with epilepsy had IL-1α mRNA levels that were five-fold higher than those of neurologically normal controls. In contrast to the epilepsy-associated increase in IL-1α mRNA in patients of both genotypes, IL-1α protein measured by Western blot showed much greater elevation of IL-1α protein in *APOE ε3,3 *patients than that in *APOE ε4,4 *patients (Figure 4C). The increased IL-1α expression in *APOE ε3,3 *patients was approximately four-fold that of *APOE ε4,4 *patients (*APOE ε3,3 = *51 ± 29 vs *APOE ε4,4 = *9 ± 4; control 6 ± 1; *P *< 0.01) (Figure 4D). These elevations in the levels of both IL-1α mRNA and protein were accompanied by an *APOE ε3,3 *genotype-specific increase in βAPP expression in epilepsy patients compared to controls (*APOE ε3,3 *= 42 ± 11 vs *APOE ε4,4 *= 20 ± 11; and control = 21 ± 7; *P *= 0.03) (Figure 4E, F).

### Aβ and ApoE deposition relative to *APOE *genotype

**Figure 5:**
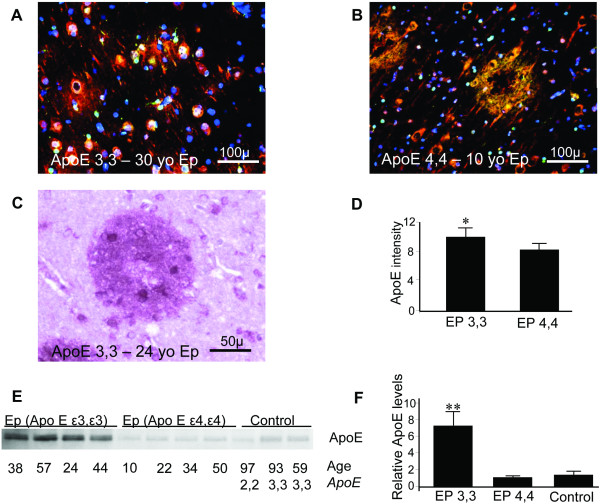
**ApoE tissue levels and Aβ plaques in epilepsy relative to APOE genotype Multiple Aβ (green) and ApoE (red) immunofluorescent plaques were present in 15 of the 52 *APOE ε3,3 *patients (colocalization of ApoE and Aβ is yellow)**. **(A) **Representative section from a 30-year-old APOE ε3,3 patient. **(B) **Representative section from the one of seven *APOE ε4,4 *patients who had Aβ plaques. **(C) **Both the shape and distribution to Aβ plaques were similar without regard to *APOE *genotype as shown in a 24-year-old *APOE ε3,3 *patient illustrated by light microscopy (ApoE immunoreactivity (brown); Aβ (magenta)). **(D) **ApoE immunofluorescent intensity was greater in tissue from those with *APOE ε3,3 *than in those with *APOE ε4,4 *genotype (*APOE ε3,3 *= 10 ± 1 vs *APOE *ε4,4 = 8.3 ± 0.8; *P *= 0.04). This difference in intensity was paralleled by increased relative tissue levels of ApoE **(E) **(*APOE ε3,3 *= 7.19 ± 1.75 vs *APOE ε4,4 *= 1.04 ± 0.06, and control = 1.27 ± 0.38; *P *< 0.001 for all comparisons) **(F)**.

We found Aβ neuritic plaques in 13 of 52 *APOE ε3,3 *patients, including one who was only 17 years old; one of the 7 *APOE ε4,4 *patients had plaques; the youngest, 10 years of age (Figure 5A-C). For this one *APOE ε4,4 *patient, the number of plaques observed was 17, which was similar to that found in four *APOE ε3,3 *patients who had Aβ plaques, numbering 11, 15, 14 and 16. Interestingly, the Aβ plaque phase in the one *APOE ε4,4 *patient was dense core neuritic while the plaques in *APOE ε3,3 *patients did not have dense cores. With our limited number of patients with *APOE ε4,4 *genotype, it is not possible to say whether a specific *APOE *genotype is associated with either a higher probability of having plaques or a genotype-related earlier age of onset for such Aβ plaques. However, epilepsy was associated with an increased probability of Aβ plaque development at a very early age. All Aβ plaques observed contained ApoE deposits as well as ApoE-immunoreactive neurites, without regard to age or gender.

### Neuronal expression of ApoE is related to *APOE *genotype

In addition to ApoE immunoreactivity in Aβ plaques and in Aβ plaque-associated neurites in tissue from epilepsy, ApoE expression was also evident in neurons in all regions examined without regard to the presence of Aβ plaques. Semi-quantitative ApoE immunofluorescence intensity in neuronal somas was elevated in those with *APOE ε3,3 *genotype relative to those with *APOE ε4,4 *genotype (*APOE ε3,3 *= 10 ± 1 vs *APOE ε4,4 *= 8.3 ± 0.8; *P *< 0.05) (Figure 5D). This modest elevation in immunofluorescence intensity was paralleled in *APOE ε3,3 *patients by a marked increase in relative tissue levels (Figure 5E) of ApoE (*APOE ε3,3 *= 7.19 ± 1.75 vs *APOE ε4,4 *= 1.04 ± 0.06, and control = 1.27 ± 0.38; *P *< 0.001) (Figure 5F).

### Synaptophysin and phosphorylated tau expression relative to *APOE *genotype

**Figure 6:**
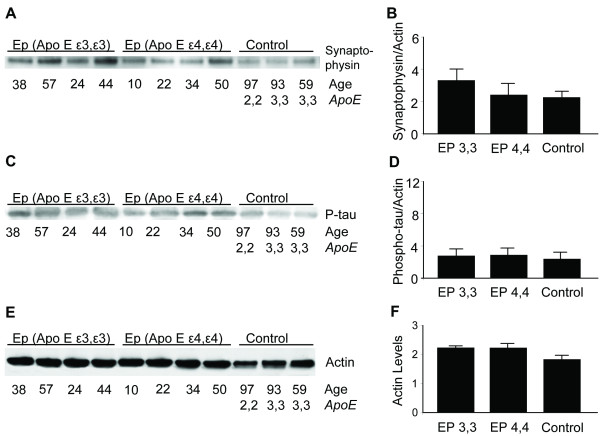
**Synaptophysin, phosphorylated tau and actin in epilepsy relative to APOE genotype An impression of similar synaptic densities in photomicrographs from each of the patient groups was confirmed by western blot analysis for the relative levels of synaptophysin compared to actin (*APOE ε3,3 *= 3**.57 ± 0.76 vs *APOE ε4,4 *= 2.62 ± 0.8 vs controls = 2.41 ± 0.36; *P *= 0.11 for all comparisons) **(A and B)**. Western blot analysis of phosphorylated tau (P-tau) **(C) **showed no differences with regard to genotype **(D)**. Western blot analysis of actin **(E) **was used to assess specific protein levels of IL-1α, βAPP, ApoE, synaptophysin, and P-tau relative to actin. Actin expression was not different with regard to APOE genotype or levels of actin in tissue from controls **(F)**.

The synaptic density in photomicrographs from each of the patient groups appeared to be similar, with somewhat higher levels in those with *APOE ε3,3 *genotype, as measured by relative levels of synaptophysin (*APOE ε3,3 *= 3.57 ± 0.76 vs *APOE ε4,4 *= 2.62 ± 0.80 vs controls = 2.41 ± 0.36; *P *= 0.11) (Figure 6A. B). As previously reported [14], phosphorylated tau immunofluorescent labeling was not elevated in any of our patients regardless of age or *ApoE *genotype. This was confirmed by Western blot analysis; the bands were faint and the measured levels were low (Figure 6C, D).

## Discussion

Tissues from patients undergoing temporal lobectomies for drug-resistant epilepsy reveal *APOE *genotype-specific links between glial and neuronal stress responses. This influence of *APOE *genotype in epilepsy appears to occur without regard to gender or age at the time of surgery. Glial activation with overexpression of IL-1 is well known to induce neuronal expression of two AD-associated, stress-related proteins ApoE and βAPP [10,15]. Connections among *APOE *genotype, epilepsy and AD have been drawn, but mechanisms by which the *APOE ε4,4 *genotype heightens intensity of neuronal damage or, conversely, how the *APOE ε3,3 *genotype may act to promote neuronal resilience remains unclear.

The numbers of neurons in temporal lobe tissue of our epilepsy patients who were either *APOE ε3,3 *or *APOE ε4,4 *genotype were similar, but there were striking differences in the indicators of degeneration in neurons, as neurons from patients with *APOE ε3,3 *were larger, appeared more normal morphologically, and had less DNA damage. These findings suggest that neurons from individuals with the *APOE ε3,3 *genotype are better able to mount appropriate and more liberal repair responses to the damaging hyperexcitability of epilepsy than are their *APOE ε4,4 *counterparts, suggesting that *APOE ε3*, but not *APOE ε4*, alleles confer resilience to host neurons no matter the type of injury. This might be inferred from studies reporting earlier onset of epilepsy, especially following traumatic brain injury in patients with *APOE ε4 *alleles [16,17].

Our finding of elevated synthesis of IL-1α in the temporal lobe of epilepsy patients compared to that in neurologically normal controls confirms an earlier report [4] of elevated IL-1α protein and accompanying glial activation and other neuroinflammatory changes. However, the association made here between this overexpression of IL-1α and beneficial effects toward enhancing neuronal resilience may help to explain, at least in part, why IL-1α elevation is necessary for neuronal survival in dorsal root ganglion cell cultures [18]. Moreover, evidence of greater neuron sparing in epilepsy patients with *APOE ε3,3 *than *APOE ε4,4 *genotype may be a case in point for genetic variation favoring typical, evolutionarily old, acute phase responses [19] of neurons to adverse stimuli, which includes elevation of IL-1α, βAPP and ApoE expression [10] and protection against DNA fragmentation.

The original report of a role for IL-1α in induction, maintenance and propagation of axonal sprouting in an experimental model of neurodegeneration [20] and an association between glial activation and sprouting of mossy fibers in epilepsy [21] is supported by our finding of somewhat elevated synaptophysin levels in combination with high numbers of neuron-associated, IL-1α immunoreactive microglia and elevation of IL-α mRNA and protein levels. In addition, the apparent elevation of synaptophysin expression noted here in immunoblots of neural tissue proteins from our epilepsy patients compared to that from our neurologically and neuropathologically normal controls may be explained if, as previously noted in animal models of epilepsy, [21,22] there is neuronal sprouting in epilepsy patients.

Amyloid-β plaques are obligatory for the diagnosis of AD and are most prominent in the elderly. In contrast, Aβ plaques in epilepsy, as shown here and as reported in about 10% of cases [2], are evident at young ages. For instance among our patients, a 10-year-old patient had Aβ/ApoE immunoreactive plaques in a distribution similar to that noted in temporal lobes of Alzheimer patients. The presence of plaques at such early ages suggests that they are harbingers of impending neurodegeneration and AD. Although the number of plaques was similar in tissue from our patients without regard to *APOE *genotype, in our one *APOE ε4,4 *patient the developmental phase of Aβ plaques appeared to be advanced relative to those observed in our *APOE ε3,3 *patients -- our *APOE ε4,4 *patient had dense core neuritic Aβ plaques, while such dense core plaques were not found among the plaques observed in our *APOE ε3,3 *patients. This observation is consistent with the possibility that the phase of Aβ plaque progression is accelerated in those with *APOE ε4,4 *genotype and supports the findings of Marz *et al.*, regarding the role of *APOE *genotype in the onset of Aβ plaque pathology and the presence of dense core plaques [23].

Alzheimer's patients are more likely to have seizures than are those in the general population [24]. This, together with our findings and the previously reported preferential occurrence of seizures in younger Alzheimer patients [25], supports a suggested relationship between the high levels of Aβ in the brains of epilepsy patients [26] and increased risk for development of AD. These findings are consistent with the idea that AD-related neuronal stress and its sequelae, including excess neuronal βAPP and ApoE expression and glial activation with elevated cytokine expression, combined with known IL-1-driven elevation of neuronal and glial glutamate production contribute to the hyperexcitability of epilepsy [9]. Moreover, these findings, together with evidence from our epilepsy patients, suggest that ApoE genotype, in particular *APOE ε4,4 *may favor rapidity of disease progression as well as risk for associated memory disturbances. Conversely, a better understanding of mechanisms by which *APOE ε3 *alleles confer the neuronal protection shown here may facilitate development of therapeutic strategies toward improving outcomes for epilepsy patients, as well as patients with other neuronal distresses.

## Conclusion

The most striking aspect of this work is that our findings illuminate the "other" side of the *APOE *genotypic equation in showing ways in which *APOE ε3 *alleles may act to preserve important aspects of neuronal abilities to mount appropriate, beneficial stress responses to hyperexcitability, neuroinflammation and neuronal DNA damage. In addition, our findings are consistent with the idea that as neurons with *APOE ε4 *alleles are less resilient to the chronic excitation of epilepsy and more susceptible to DNA damage, patients who carry *APOE ε4 *alleles are at greater risk of developing AD than are those with *APOE ε3 *alleles. Moreover, our findings are in accord with the possibility that epilepsy-related neuropathological changes, such as increases in the levels of Aβ peptides, contribute to propagation of epileptiform activity in adjacent neurons and furtherance of neuropathological changes and the risk of AD [26].

## Abbreviations

Aβ: amyloid β; AD: Alzheimer's disease; ApoE: apolipoprotein E; au: arbitrary units; βAPP: amyloid β precursor protein; IL: interleukin; TUNEL, terminal deoxynucleotidyl transferase dUTP nick end labeling.

## Competing interests

The authors declare that they have no competing interests.

## Authors' contributions

OA conducted and helped with the design of all experiments, with interpretation of the data and writing of the manuscript. REM conducted all neuropathological evaluations and contributed to interpretation of results and writing of the manuscript. FB was the neurosurgeon who provided the tissue and reviewed the writing. WSTG designed the study with OA, verified and helped with interpretation of the data, and contributed to the writing. All authors read and approved the final manuscript.

## Pre-publication history

The pre-publication history for this paper can be accessed here:

http://www.biomedcentral.com/1741-7015/10/35/prepub
